# A review of pathogenic airborne fungi and bacteria: unveiling occurrence, sources, and profound human health implication

**DOI:** 10.3389/fmicb.2024.1428415

**Published:** 2024-09-19

**Authors:** Amran A. Q. A. Al-Shaarani, Lorenzo Pecoraro

**Affiliations:** ^1^College of Pharmaceutical Science & Moganshan Research Institute at Deqing County, Zhejiang University of Technology, Hangzhou, China; ^2^School of Pharmaceutical Science and Technology, Tianjin University, Tianjin, China

**Keywords:** human health, airborne microbes, respiratory ailments, allergies, risk exposure, environmental sources, indoor and outdoor environments, pathogenic microorganisms

## Abstract

Airborne fungi and bacteria have been extensively studied by researchers due to their significant effects on human health. We provided an overview of the distribution and sources of airborne pathogenic microbes, and a detailed description of the detrimental effects that these microorganisms cause to human health in both outdoor and indoor environments. By analyzing the large body of literature published in this field, we offered valuable insights into how airborne microbes influence our well-being. The findings highlight the harmful consequences associated with the exposure to airborne fungi and bacteria in a variety of natural and human-mediated environments. Certain demographic groups, including children and the elderly, immunocompromised individuals, and various categories of workers are particularly exposed and vulnerable to the detrimental effect on health of air microbial pollution. A number of studies performed up to date consistently identified *Alternaria*, *Cladosporium*, *Penicillium*, *Aspergillus*, and *Fusarium* as the predominant fungal genera in various indoor and outdoor environments. Among bacteria, *Bacillus*, *Streptococcus*, *Micrococcus*, *Enterococcus*, and *Pseudomonas* emerged as the dominant genera in air samples collected from numerous environments. All these findings contributed to expanding our knowledge on airborne microbe distribution, emphasizing the crucial need for further research and increased public awareness. Collectively, these efforts may play a vital role in safeguarding human health in the face of risks posed by airborne microbial contaminants.

## 1 Introduction

Airborne microorganisms, particularly bacteria and fungi, have been identified as a possible source of significant hazards to human health, potentially leading to a spectrum of pathologic conditions ranging from infectious diseases to allergic and toxic reactions (Pastuszka et al., 2000; Hedayati et al., 2005; Simon-Nobbe et al., 2008; Fernstrom and Goldblatt, 2013). When human individuals breathe in either indoor or outdoor air, they are exposed to a substantial number of microbial cells, some of which can act as pathogens or trigger allergic conditions (Barberán et al., 2015a). Therefore, exposure to airborne microorganisms represents a serious risk for human health, resulting in various respiratory disease and infections (Shams-Ghahfarokhi et al., 2014). This risk is further amplified by the extensive dispersion potential of these microorganisms through air currents, enabling their inhalation, ingestion, or contact with individuals who have not had direct exposure to the original source of infection (Fernstrom and Goldblatt, 2013). Fungi are an essential part of airborne microbial communities, given their abundance and wide distribution in a number of environmental sources, such as soil, water and decaying vegetation (Horner et al., 2004; Sen and Asan, 2009; Kalyoncu, 2010; Chakrabarti et al., 2012). The attention on airborne fungal contaminants has dramatically increased together with the evidence of the health hazards directly caused by the fungal spores themselves or by the metabolites released by the fungal particles present in the air that people inhale (Shams-Ghahfarokhi et al., 2014). Apart from the potential for fungal infections ranging from mild to life-threatening, including those acquired in healthcare settings, the health impacts of fungal bioaerosols encompass allergenic, toxigenic and inflammatory effects (Fischer and Dott, 2003; Hedayati et al., 2005; Simon-Nobbe et al., 2008). Fungal spores have the capacity to act as reservoirs for significant quantities of toxic secondary metabolites, specifically mycotoxins, thereby presenting a potential health hazard when inhaled via airborne bioaerosols and dust (Araujo and Cabral, 2010; Shams-Ghahfarokhi et al., 2014). Under special circumstances, the release of pathogenic bioaerosols into the air may also occur as a discharge from the respiratory tract of infected individuals, during routine activities, such as talking, sneezing, coughing, and breathing (Han et al., 2013; Prussin and Marr, 2015; Madhwal et al., 2020), consequently contributing to an increased risk of exposure, particularly in public spaces characterized by intense human presence, such as subway and train stations, places of worship, market areas, and hospitals (Dong and Yao, 2010; Alananbeh et al., 2017; Madhwal et al., 2020). In particular, it is noteworthy that nosocomial infections transmitted through airborne routes can further amplify the likelihood of wound infections within specific healthcare settings (Fleischer et al., 2006). It has been hypothesized that high concentrations of fungal volatile organic compounds in outdoor environments have the ability to impact human health by inducing symptoms like headaches, fatigue, and irritation of the eyes, throat, and nose (Rolle-Kampczyk et al., 2000; Araujo and Cabral, 2010). Extensive research efforts have been dedicated to the analysis of fungal communities in indoor and outdoor environments (Wu et al., 2000; Shelton et al., 2002; Hedayati et al., 2005; Muafa et al., 2024). Numerous studies have shed light on the identity and concentration of dominant fungal genera in the atmosphere, including *Cladosporium*, *Alternaria*, *Aspergillus*, and *Penicillium*. Among them, *Cladosporium* has emerged as the dominant taxon responsible for the presence of allergic fungal spores in various regions (Kalyoncu, 2010; Chakrabarti et al., 2012; Lang-Yona et al., 2012; Fernstrom and Goldblatt, 2013; Sepahvand et al., 2013). As far as airborne bacteria are concerned, these microorganisms exhibit a wide distribution within the lower atmosphere, encompassing a vast range of habitats. Pathogenic bacteria are of particular importance from the medical point of view, since they have significant effects on human health (Burrows et al., 2009; Bowers et al., 2011; Fan C. et al., 2019; Hu et al., 2020). These pathogens have a greater ability to grow and survive in harsh environments compared to non-pathogenic bacteria. For instance, under extreme air pollution, pathogenic bacteria can increase their relative abundance, thus posing significant health hazard to humans (Lee and Lee, 2016; Liu et al., 2018). Bacteria can be found in the atmosphere either as single cells or in association with various particles such as spores, soil, dust, leaves, and other microorganisms (Tong and Lighthart, 2000; Maron et al., 2005; Maki et al., 2008). Once bacteria originated from different sources enter the air environment, they can be transported upward by convective air currents, and due to their small size, they can persist in the atmosphere for a long time (Smets et al., 2016). Notably, bacteria have been observed traveling across continents, especially when associated with dust storms originating from deserts or areas affected by drought (Kellogg and Griffin, 2006; Polymenakou et al., 2008; Lim et al., 2011; Hara and Zhang, 2012; Barberán et al., 2014). This ability of bacteria to be transported over long distances through the atmosphere may results in the potential spreading of certain diseases to different regions (Hara and Zhang, 2012; Fan C. et al., 2019). The airborne transmission of bacteria may involve various well-known pathogenic genera, such as *Neisseria*, *Staphylococcus*, and *Corynebacterium*, which have both pro-inflammatory and pathogenic properties. *Neisseria*, *Corynebacterium*, and *Bacillus* have been reported as prominent causative agents of anthrax, diphtheria, and meningitis (Tettelin et al., 2000; Klee et al., 2010; Fan C. et al., 2019), while the species *Pseudomonas aeruginosa* is a notable example of a pathogenic bacterium that significantly contributes (10–20%) to infections in hospital settings (Fan C. et al., 2019). Overall, given the tremendous impact of airborne fungi and bacteria on human health, which results in the insurgence of various infectious diseases, allergic reactions, and toxic effects, it is crucial to achieve a comprehensive understanding of microbial community diversity and structure in different air environments. The primary objective of this review is to provide valuable insights into the spatial occurrence and distribution of airborne pathogenic microbes in both outdoor and indoor environments, with a particular focus on their adverse effects on human well-being. Our aim is to provide comprehensive information on the human health hazard created by the presence of microorganisms in the air, which could help in the development of effective strategies for mitigating the adverse effects associated with microbial pollution.

## 2 Sources of airborne fungi

Airborne fungi can be found in different indoor and outdoor air environments, originating from a variety of natural sources, including vegetation, soil, dust, water, and human activities, such as agriculture, composting, construction, demolition, and other occupations (Gilbert and Duchaine, 2009; Pecoraro et al., 2013; Qi et al., 2020). Understanding the sources and properties of airborne fungi is critical for determining their potential influence on human health and implementing suitable mitigation strategies. Depending on factors including vegetation type, geographic location, and weather conditions, the diversity and concentration of airborne fungi might vary between different places (Awad, 2005). Numerous studies suggest that vegetation constitutes a main source of fungal particles that are released into the surrounding air environments (Awad, 2005; Lymperopoulou et al., 2016; Pecoraro et al., 2012, 2021). It has been shown that various microbes colonize the phylloplane (Mercier and Lindow, 2000), which plays a significant role in the presence of airborne fungi, while high concentrations of fungal spores in the air may be caused by the extensive microbial covering of leaves (Qi et al., 2020). According to a study conducted by Qi et al. (2020), focusing on urban and mountainous regions of Xi’an City, China, the main local source of airborne fungi was the surface of the leaves across all seasons, with a significantly lower contribution from the soil. Humans and animals, under particular pathologic conditions, can also be a source of fungi. For instance, the skin-associated fungal genus *Microsporum* has been predominantly found in animal and human hair (Qi et al., 2020), having the ability to induce various human adverse health conditions and to increase the chances of developing psoriasis (Machado et al., 2005; Qi et al., 2020). Such fungal species, commonly associated with the human skin, can be disseminated into the air upon skin shedding (Prussin and Marr, 2015; Findley et al., 2013). A number of both organic and inorganic environmental sources, such as dust and water, may also contribute to the presence and abundance of fungi in the air. In a study conducted at five different subway stations in Seoul City, the analyzed 12 stagnant water and five settled dust samples exhibited significantly high fungal concentrations and were considered an important potential source of airborne fungi (Cho et al., 2006). A large body of literature reported that outdoor air can be a source for different indoor airborne fungi (Adams et al., 2013; Barberán et al., 2015a). A study conducted in one of the Singapore library buildings revealed that the concentrations of indoor fungi were roughly 50 times lower than those found in outdoor air (Goh et al., 2000). However, airborne fungi can also be generated from indoor sources such as sinks and shower faucets (Prussin and Marr, 2015), which have been reported to aerosolize *Aspergillus* spp. and *Fusarium* spp. in hospital settings (Anaissie et al., 2002). Residential showers have also been found to produce bioaerosols containing *Alternaria alternata*, *Penicillium* spp., *Cladosporium* spp., *Acremonium* spp., and *Paecilomyces variotii* (Prussin and Marr, 2015).

## 3 Health implications of exposure to airborne fungi

Airborne fungi pose significant health concerns for humans worldwide (Wu et al., 2000; Atya et al., 2019; Tiew et al., 2020; Fisher et al., 2022; Nageen et al., 2023; Kasprzyk et al., 2021). A number of airborne fungal species cause various health problems, including allergic reactions, infectious diseases, toxicosis reactions, respiratory ailments and pathologic conditions like aspergillosis, asthma, hypersensitivity, and pneumonitis (Wu et al., 2000; Górny et al., 2002; Patel et al., 2018; Odebode et al., 2020; Sio et al., 2021; Pashley and Wardlaw, 2021; Nageen et al., 2023). The number of people, particularly children, affected by fungal-related disorders is on the rise (Makri and Stilianakis, 2008). As airborne spores of diverse fungal species disperse in the atmosphere, they contribute to air pollution, which can have potential implications for human health (Shelton et al., 2002; Nageen et al., 2021). In fact, humans are exposed to a substantial number of fungal spores on a daily basis, inhaling between 1,000 and 10 billion spores per day (Gao et al., 2022). This continuous and significant exposure to fungal bioaerosol emphasizes the necessity of understanding the link between airborne fungi and human health, particularly as far as respiratory problems, such as asthma, are concerned. Numerous studies have consistently revealed a strong association between exposure to airborne fungi, asthma and sensitization to fungal allergens. For instance, in a study conducted in Tucson, Arizona, a significant correlation between severity of asthma and positive skin tests for *Alternaria* mold was observed in a group of individuals (Martinez et al., 1997). In Sweden and Switzerland, 3–4% of asthma patients showed positive reactivity to fungal allergens, whereas in the United States, the proportion rose significantly to 80% (Kasprzyk, 2008; D'amato and Spieksma, 1995). In another study, a positive skin test for at least one of the fungal species (*Alternaria tenuis*, *Epicoccum nigrum*, *Cladosporium cladosporioides*, and *Helminthosporium maydis*) was found in 54% of patients admitted to the intensive care unit for asthma (Black et al., 2000). Sensitization to *Alternaria alternata* or *Cladosporium herbarum* was linked to severe asthma in a number of European nations, as well as in New Zealand, Australia, and United States, according to a study involving 1,132 patients (Zureik et al., 2002). In England, UK, adult patients with severe asthma showed a higher prevalence of positive skin tests for different molds, including *A. fumigatus*, *C. herbarum*, *A. alternata*, *Penicillium notatum*, and the yeast *Candida albicans*, compare to individuals with moderate or mild asthma (O'Driscoll et al., 2005).

## 4 Common pathogenic airborne fungi

### 4.1 Alternaria

*Alternaria*, one of the most common fungi in the atmosphere, holds particular importance in the field of aerobiology due to its association with various human health conditions. Among the numerous *Alternaria* species present in the air, *A. alternata* was described as one of the most abundant fungi in indoor environments in the United States (Woudenberg et al., 2015; Nascimento et al., 2019). Alongside *Aspergillus* and *Cladosporium*, *Alternaria* has garnered attention as a dominant fungal genus in both indoor and outdoor environments of various countries (Sharma et al., 2011; Fang et al., 2013; Nascimento et al., 2019). *Alternaria* fungi are closely related to the development of immunoglobulin E (IgE)-mediated respiratory diseases (Khosravi et al., 2009; Fuiano et al., 2012). Downs et al. (2001) observed a significant correlation between the concentration of *Alternaria* airborne fungi and the increase in airway responsiveness and respiratory symptoms. The literature consistently supports the association of *Alternaria* species with various health issues, including asthma, allergic rhinosinusitis, oculomycosis, hypersensitivity pneumonitis, allergic bronchopulmonary mycosis, and skin infections (Pulimood et al., 2007; Pastor and Guarro, 2008; Nascimento et al., 2019; Mohammad and Khalil, 2022). Pulimood et al. (2007) notably observed that individuals sensitive to *Alternaria* allergens have exacerbated asthma symptoms after exposure to this group of fungi. The presence of *Alternaria* spores in the air has been linked to an increase in hospitalization rates among children and adolescents with asthma, emphasizing the potential impact of this fungal genus on public health (Tham et al., 2017; Nascimento et al., 2019).

### 4.2 Cladosporium

*Cladosporium* fungi have been linked to a variety of health problems, including pulmonary diseases, cutaneous infections, and phaeohyphomycosis (Castro et al., 2013; Nascimento et al., 2019). In particular, the genus *Cladosporium* has been recognized as a main air contaminant in hospital settings (Maldonado-Vega et al., 2014; Chaivisit et al., 2018; Nascimento et al., 2019). In a study conducted in Barcelona, Spain, *Cladosporium* was the prevalent fungal genus in nasal microbiota samples from both allergic and healthy individuals, with *C. herbarum* and *C. cladosporioides* being the dominant species. In the latter study, antigen-specific IgE and histamine release tests on patients with bronchial asthma and/or rhinosinusitis revealed that 26% of the tested individuals were sensitized to *Cladosporium* (Sellart-Altisent et al., 2007). The link between human health problems and *Cladosporium* fungi, including the development of allergies and asthma, has been largely documented. A study conducted in Poland reported on peaks of intensity for *Cladosporium*-related allergic symptoms occurring in summer and autumn (Bednarz et al., 2016). *Cladosporium cladosporioides* is known to cause subcutaneous phaeohyphomycosis (Gugnani et al., 2006; Sang et al., 2012). Castro et al. (2013) reported on a notable case of a 27-year-old female immunocompetent chemical engineer employed at a cork company in Portugal who suffered from a pulmonary infection caused by *C. cladosporioides*.

### 4.3 Penicillium

*Penicillium* fungi are mainly pathogenic to individuals with immunocompromised systems, resulting from an initial HIV infection or various medical treatments (Barcus et al., 2005; Nascimento et al., 2019). The first human infection caused by this fungal genus was recorded in 1973, when *Penicillium marneffei* was isolated from the spleen of a patient with Hodgkin’s disease (Nascimento et al., 2019), which highlighted the potential risk of exposure to this pathogenic fungus, particularly for persons with health problems. In addition to its pathogenic properties as agent of respiratory infections, wheezing, and allergic reactions, such as allergic alveolitis and allergic asthma, *Penicillium* has garnered considerable scientific attention due to its classification as one of the most common allergenic fungal taxa in indoor and outdoor environments (Goodman et al., 2011; Abdel Hameed et al., 2009; Pashley and Wardlaw, 2021; Hughes et al., 2022). Multiple studies have demonstrated that *Penicillium* fungi cause allergic reactions, particularly asthma (Bundy et al., 2009; Knutsen et al., 2012). *Penicillium* has been explicitly linked to an increase in peak expiratory flow variability among young asthmatic patients (Bundy et al., 2009). Exposure to a significant amount of *Penicillium* spores was found to lead to the development of both immediate and delayed asthma symptoms in sensitive people (Knutsen et al., 2012; Al-Shaarani et al., 2023). This implies that exposure to *Penicillium* can cause respiratory symptoms and exacerbations, which may have an effect on the exposed individuals’ lung function and general respiratory health (Gent et al., 2012; Baxi et al., 2016). It also emphasizes the need for efficient ways to reduce indoor exposure to *Penicillium*, especially in environments where people with respiratory disorders spend a lot of time. Therefore, the presence and abundance of *Penicillium* in indoor and outdoor air environments have received substantial interest from researchers. For instance, an investigation conducted in urban outdoor environments in Tianjin, China, revealed that *Penicillium* was the third most abundant fungal genus recorded (Nageen et al., 2023), while a study conducted in a research and teaching building of Tianjin University (Tianjin, China) reported that *Penicillium* was the fourth most diverse fungal genus in the various analyzed indoor and outdoor building environments. The latter findings underscored the extensive distribution of *Penicillium* in the studied environments, and the potential risk of exposure for people to this pathogenic fungal genus.

### 4.4 Fusarium

*Fusarium* species commonly thrive and grow in agricultural settings and have the ability to produce toxic secondary metabolites called mycotoxins that contaminate crops (e.g., barley, rice, and corn) during growth, harvesting, transportation and storage stages (Goswami and Kistler, 2004; Antonissen et al., 2014; Shams-Ghahfarokhi et al., 2014). Exposure to these mycotoxins can have negative effects on human health, including the potential disruption of the immune system and the damage of the intestinal epithelium (Maresca, 2013; Liew and Mohd-Redzwan, 2018; Nafis et al., 2024). Notably, *Fusarium* has been implicated in a wide array of infections, particularly among immunocompromised individuals (Nascimento et al., 2019). The available literature indicates that exposure to this fungal genus has the potential to induce allergies that may contribute to the development of asthma in susceptible individuals (Khosravi et al., 2012). Hoff et al. (2003) effectively isolated and described an allergen produced by *Fusarium culmorum*, which was reactive in 44% of sera from people who were at risk for allergies (Hoff et al., 2003). Furthermore, species of *Fusarium* are increasingly recognized as novel human pathogens, commonly isolated from ocular tissues and less frequently from skin blood and nails, with a higher incidence seen in immunocompromised hematological patients (Tupaki-Sreepurna and Kindo, 2018).

### 4.5 Aspergillus

*Aspergillus* fungi, commonly found in various environments, include approximately 20 distinct species capable of inducing diseases in human hosts (Dagenais and Keller, 2009; Nageen et al., 2023). The spores of this fungal species have been found to predominate in the air during the fall and winter seasons in the United Kingdom, raising concerns on the possibility of human exposure (Millington and Corden, 2005). Among *Aspergillus* species, *A. fumigatus* is one of the most common human pathogenic fungi, responsible for over 90% of all cases of invasive aspergillosis (IA), as well as for life-threatening lung infections and allergic bronchopulmonary aspergillosis (Dagenais and Keller, 2009; Nafis et al., 2023). IA have a death rate of 60–90% because of difficulties in diagnosis, lack of effective anti-fungal therapies, and the rise of drug-resistant strains. The severity of these infections largely depends on the immune system and general health conditions of susceptible individual (Singh and Paterson, 2005; Vödisch et al., 2009). The small size of *A. fumigatus* conidia allows them to move deep into the respiratory system, colonizing the alveoli as the primary step of systemic *Aspergillus* infections (McCormick et al., 2010). Besides, *A. fumigatus*, together with *A. flavus* and *A. niger*, have the capacity to infect tissues not only within the respiratory system, but also eyes, skin, central nervous system, and nails (Patterson et al., 2016; Tsai et al., 2019; Lai et al., 2020; Jing et al., 2022). *Aspergillus flavus* is an opportunistic fungal pathogen that can colonize the respiratory tract and cause invasive aspergillosis, fatal infections, especially in immunocompromised patients (Curbelo et al., 2015; Lu et al., 2022; Nafis et al., 2024). Previous studies have also reported that inhalation or ingestion of *A. flavus*, known for its production of aflatoxin B, can result in the development of lung cancer in humans (Georggiett et al., 2000; Marchese et al., 2018). *Aspergillus niger* is recognized as a pathogenic fungus and a potent allergen, associated with lung infections. It can also cause invasive aspergillosis, systemic mycosis, cutaneous infections, allergic bronchopulmonary diseases, and, in some cases, pneumonia (Bulpa et al., 2007; Person et al., 2010; Marr et al., 2002).

## 5 Sources of airborne bacteria

Airborne bacteria originate from various sources, including dust, soil, plants, water bodies, animals, and humans (Bowers et al., 2011; Fan X. Y. et al., 2019; Sun et al., 2018; Mu et al., 2020). Various studies conducted worldwide have highlighted the importance of soil and leaf surfaces as the main contributors to bacterial presence in the lower atmosphere (Brodie et al., 2007; Bowers et al., 2011; Gao et al., 2017; Ruiz-Gil et al., 2020). For instance, a study conducted in mountainous and urban areas of Xi’an City, China, suggested that the primary sources of airborne bacteria in summer and autumn are soil and leaf surfaces (Mu et al., 2020). Another study indicated that airborne bacterial communities are similar to those found in soil (Brodie et al., 2007), possibly due to the influence of land-use type, as different habitats act as sources of bacteria that can be released into the air, potentially affecting the overall diversity and concentration of airborne microbes (Després et al., 2007; Redford et al., 2010). Deserts and dry areas have been previously reported as major sources of aerosolized bacteria attached to dust particles that are abundantly generated in these environments, forming aerosols capable of traveling long distances with the assistance of wind (Maki et al., 2008). Marine environments also contribute to aerosolizing bacteria through water surface, aided by sea sprays generated by high winds and breaking waves (Graham et al., 2018; Ruiz-Gil et al., 2020). Airborne bacteria can also originate from anthropic activities and human-mediated environments, including wastes from hospitals, houses, and pet feces in urban environments, as well as agricultural practices, livestock farming, and waste treatment, such as wastewater management and composting (Ruiz-Gil et al., 2020) in rural areas. In particular, composting involves thermophilic actinomycetes, which play a fundamental role in the degradation process, but can also trigger allergic responses in humans, including asthma and hypersensitivity pneumonitis (Sharma et al., 2014). Dog feces, especially in urban environments, can represent an unexpected important source of bacteria in the atmosphere, especially during the winter season, according to a study conducted in the Midwestern United States (Bowers et al., 2011). Plants and animals (e.g., dogs and cats) have also been observed to release microorganisms into the air, making a notable contribution to the indoor bacterial flora (Barberán et al., 2015b; Xie et al., 2021). Wastewater treatment plants are another important source of bacterial bioaerosols. Many studies consistently demonstrated that the microorganisms present in these wastewater facilities contribute notably to the overall composition of airborne bacterial communities in surrounding areas (Degois et al., 2017; Yang et al., 2019; Xie et al., 2021). Metalworking fluids used in engineering environments may show the presence of Gram-negative bacteria. These bacteria produce endotoxins, lipopolysaccharide (LPS) molecules located in the outer envelope of the bacterial cell walls, also known as pyrogens due to their ability to induce fever. Exposure to endotoxins can lead to flu-like symptoms, including inhalation fever, thus making it essential to address the impact of such molecules on human health, particularly in occupational settings where metalworking fluids are prevalent (Passman and Küenzi, 2020). Human bodies and daily activities may contribute to the release of substantial amounts of bacterial aerosols, which can result in potential health risks and contribute to outdoor and indoor air pollution (Xie et al., 2021). Specifically, different types of bacteria inhabit various internal and external parts of the human body, such as skin and digestive tract, which harbor approximately 10^12^–10^14^ microbial species (Costello et al., 2009; Xie et al., 2021). Consequently, humans have become the most dominant source of bacterial bioaerosols, particularly in indoor environments, directly influencing the composition and structure of airborne microbial flora (Hospodsky et al., 2015). *Legionella pneumophila* is commonly present in aquatic environments, including both natural reservoirs, such as rivers and lakes, as well as human-made settings, like air-conditioning systems, cooling towers, humidifiers, and public showers (Graells et al., 2018; Sánchez-Parra et al., 2019). This highly adaptable bacterium, which exhibits resilience across a wide range of temperatures, is known to cause Legionnaires’ disease, a severe form of pneumonia, and Pontiac fever, a type of legionellosis that does not involve pneumonia but has been linked to many cases of Legionnaires’ disease in the US and Europe (Blatny et al., 2011; Van Heijnsbergen et al., 2015). *Legionella* bacteria are primarily transmitted through the inhalation of aerosols containing the microbes, which leads to the development of severe respiratory infections, particularly in individuals with weakened immune systems (Allegra et al., 2016).

## 6 Health implications of exposure to airborne bacteria

As the prevalent microorganisms in the atmosphere, bacteria play an important role in shaping ecological balance and affecting human health (Hu et a., 2020). Airborne bacteria pose health risks to both residents and workers in different environments, potentially causing allergies, respiratory infections, and other systemic diseases (Yang et al., 2019; Passi et al., 2021). Inhaling pathogenic airborne bacteria can lead to various diseases and allergic reactions, such as pneumonia, asthma, rhinitis, and pharyngitis, especially in children and elder individuals (Passi et al., 2021). Different bacterial species have the ability to target specific organs within the human body, inducing various infections (Doron and Gorbach, 2008). For instance, *Staphylococcus aureus*, which is commonly found on the skin and mucous membranes, can trigger soft tissue and skin infections (Dayan et al., 2016). Besides, this bacterial species has the potential to disseminate throughout the bloodstream, causing infections in different body sites including lungs, heart valves, and abdomen (Doron and Gorbach, 2008; Dayan et al., 2016). *Neisseria meningitidis* primarily targets lungs and meninges, causing pneumonia and meningitis, respectively (Doron and Gorbach, 2008). Bacterial presence in the air is influenced by the ability of these microorganisms to colonize and grow in various liquids and surfaces (Gonzalez-Martin, 2019). Under suitable nutritional and physical conditions, bacteria can be released into the air, which enables them to be aerosolized and easily inhaled by the exposed individuals (Gonzalez-Martin, 2019). *Acinetobacter* species play a crucial role as causative agents of nosocomial infections in healthcare settings (Hwang and Park, 2014). For instance, *A. lwoffii* is a common Gram-negative bacterium capable of causing bacteremia, particularly in immunocompromised patients (Guo et al., 2021). *Bacteroides fragilis* is an opportunistic human pathogen that can cause intra-abdominal, skin, soft tissue, and postoperative wound infections (Guo et al., 2021). *Streptococcus pneumoniae* is a common pathogenic agent that can induce pneumonia and meningitis, particularly in children (Mehr and Wood, 2012). Different species of *Streptococcus* have been known to induce various infections and pathological conditions, such as acute respiratory distress syndrome, bacteremia, shock, neonatal sepsis, and meningitis (Mehr and Wood, 2012; Guo et al., 2021). *Prevotella*, a common Gram-negative bacterial genus found in the human oral cavity, can lead to some periodontal diseases (Ibrahim et al., 2017; Du et al., 2018). *Erysipelothrix* is a group of Gram-positive bacteria that has been reported as a cause of erysipelas in humans and swine (Du et al., 2018). *Enterobacter* is a pathogenic Gram-negative bacterial taxon that is predominantly isolated from patients in intensive treatment units and has the ability to cause bacteremia and other infections (Okatani et al., 2000; Du et al., 2018). *Rickettsia* species have been reported as causative agents of different human diseases, including Brill-Zinsser disease, spotted fever, and epidemic typhus (Du et al., 2018).

## 7 Common pathogenic airborne bacteria

Bacterial communities exhibit spatial and temporal variations depending on many factors such as seasonal changes, weather and environmental conditions, and human activities, which contribute to regional disparities (Bertolini et al., 2013; Zhang et al., 2022). These variations may result in different diseases caused by the varying microbes in different regions and periods of the year (Table 1). A comparison of bacterial community structures at phylum and genus levels across different regions revealed distinct variations (Zhang et al., 2022). At phylum level, Proteobacteria emerged as the predominant group in studies conducted in Toyama, Japan (Tanaka et al., 2019), while Actinobacteria and Firmicutes were found to be the dominant groups in Beijing and Jinan, respectively (Wang et al., 2015; Xu et al., 2017). At genus level, in different studies conducted in China, it was reported that *Sphingomonas* was the most abundant genus in Urumqi city (Gou et al., 2016), while in Qingdao, Jinan, and Xi’an the most dominant genera were *Acinetobacter*, *Lactococcus*, and *Pseudomonas*, respectively (Wang et al., 2015; Wang et al., 2016; Xu et al., 2017). In Hong Kong, a variety of human pathogenic bacterial genera were detected in the air, including *Legionella*, *Shigella*, *Pseudomonas*, *Staphylococcus*, *Streptococcus*, and *Salmonella* (Woo et al., 2013). *Sphingomonas* was the dominant airborne bacterial genus in a study conducted in Heraklion, Greece (Polymenakou et al., 2008). A research carried out in Po Valley (Italy), one of the most urbanized and polluted regions in Europe, showed that *Staphylococcus* and *Sphingomonas* were the dominant airborne pathogenic bacteria (Innocente et al., 2017). In a study conducted in Beijing, the bacterial composition of PM2.5, analyzed at different seasons and air pollution levels, revealed the presence of five pathogenic taxa including *Streptococcus*, *Prevotella*, *Rickettsia*, Enterobacteria, and *Erysipelothrix* (Du et al., 2018). Various studies carried out in Europe have consistently indicated that Gram-positive cocci bacteria, particularly species of *Staphylococcus* and *Micrococcus*, are commonly found in indoor air environments, whereas some Gram-negative bacteria, including the Pseudomonadaceae family and *Aeromonas* species, are often present but in lower abundance. Similarly, in a study conducted in the United States, Gram-positive cocci bacteria were also reported as prevalent in the indoor and outdoor environments analyzed in a large building consisting of 100 offices (Tsai and Macher, 2005).

**Table 1:** Distribution of common pathogenic bacterial communities across different regions.

Study’s region	Year	Dominant bacteria	Classification	Gram	Pathogenicity	Reference
Lisbon, Portugal	2022	*Escherichia coli*	Species	Negative	Hospital-acquired infections in immunocompromised individuals.	Monteiro et al. (2022)
		*Pseudomonas aeruginosa*				
		*Staphylococcus* spp.		Positive		
		*Staphylococcus aureus*				
		*Micrococcus luteus*				
Heraklion, Greece	2008	*Sphingomonas*	Genera	Negative	Nosocomial infections.	Polymenakou et al. (2008)
Xinjiang, China	2016	*Acinetobacter*			Lung infection and pneumonia	Gou et al. (2016)
Guelph, Canada	2011	*Fusobacterium nucleatum*	Species		Various inflammatory conditions and infectious diseases, including lung and urinary tract infections.	Allen-Vercoe et al. (2011)
Singapore	2011	*Pseudomonas aeruginosa*			Respiratory and gastrointestinal infections	Saeidi et al. (2011)
Boston, USA	2008	*Acinetobacter baumannii*			Community-acquired and hospital-acquired pneumonia and bloodstream infections	Peleg et al. (2008)
Marseille, France	2015	*Enterobacter aerogenes*			Hospital-acquired infections	Davin-Regli and Pagès (2015)
		*Enterobacter cloacae*				
Chicago, USA	2012	*Stenotrophomonas maltophilia*			Primarily linked to respiratory infections	Brooke (2012)
Xi’an, China	2019	*Neisseria*	Genera		Significant contributors to Diphtheriae, meningitis, and anthrax	Fan C. et al. (2019)
		*Corynebacterium*		Positive		
		*Bacillus*				
Beijing, China	2018	*Streptococcus*			Infections associated with bacteremia, shock, acute respiratory distress syndrome, meningitis, and neonatal sepsis	Du et al. (2018)
Birmingham, England	2004	*Corynebacterium diphtheriae*	Species		Causative agent of diphtheria	Dover et al. (2004)
Oklahoma, United State	2001	*Streptococcus pyogenes*			Pharyngitis, rheumatic fever, acute glomerulonephritis, toxic shock syndrome, impetigo, and scarlet fever	Ferretti et al. (2001)
Rabat, Morocco	2002	*Lactococcus lactis*			Cerebellar abscess	Akhaddar et al. (2002)
Colorado, United States	2017	*Staphylococcus*	Genera		Proinflammatory and pathogenic capacity	Schaeffer et al. (2017)
		*Pseudomonas*		Negative		
		*Streptococcus*		Positive		

## 8 Variation in susceptibility to airborne microbes

Susceptibility to airborne microbes, including fungi and bacteria, shows a significant variation among different human groups and populations. This susceptibility is affected by different factors, including occupational hazards, environmental conditions, and individual health status (Makri and Stilianakis, 2008). In general, infants, elderly, and immunocompromised patients, as well as individuals engaged in particular occupations (e.g., agriculture, healthcare, and construction) are at higher health risk by exposure to airborne microbes than other people (Madhwal et al., 2020). A study conducted in Hanoi, Vietnam, revealed that infants and young children were at a higher risk of developing respiratory disorders attributed to the high concentrations of airborne microbial particles in the city’s air (Luong et al., 2017). Elderly individuals could also be particularly susceptible to air pollutants exposure, primarily due to their compromised immune systems and the prevalence of underlying chronic diseases (Madureira et al., 2015). Farmers represent a category of workers highly susceptible to airborne microbes originating from dust, soil, and/or leaf surfaces, and commonly suffer from various health problems, notably allergic skin diseases, as a result of direct contact with microbes during their farming activities (Kasprzyk, 2008). They may also suffer from some respiratory diseases due to exposure to some pathogenic fungal spores (Kasprzyk, 2008). Similarly, healthcare workers are at significant risk of exposure to airborne microbes due to the nature of their daily work, which puts them in close contact with diverse patients, thus potentially resulting in the transmission of some infectious diseases through air routes (Zemouri et al., 2017; Wilson et al., 2020). Additionally, healthcare workers frequently employ instruments or intervention methods that may generate bio-aerosols, further increasing their exposure to airborne microbial hazards (Zemouri et al., 2017). Other various occupational groups, including transportation employees (e.g., traffic police and subway station workers), roadside workers (municipal and construction workers, etc.), markets and restaurants workers, as well as daily commuters, have been consistently reported in the literature as highly susceptible groups particularly exposed to bioaerosol at different sites (Kim et al., 2011; Madhwal et al., 2020; Al-Shaarani et al., 2023).

## 9 Common indoor versus outdoor airborne fungi and bacteria

**Figure 1:**
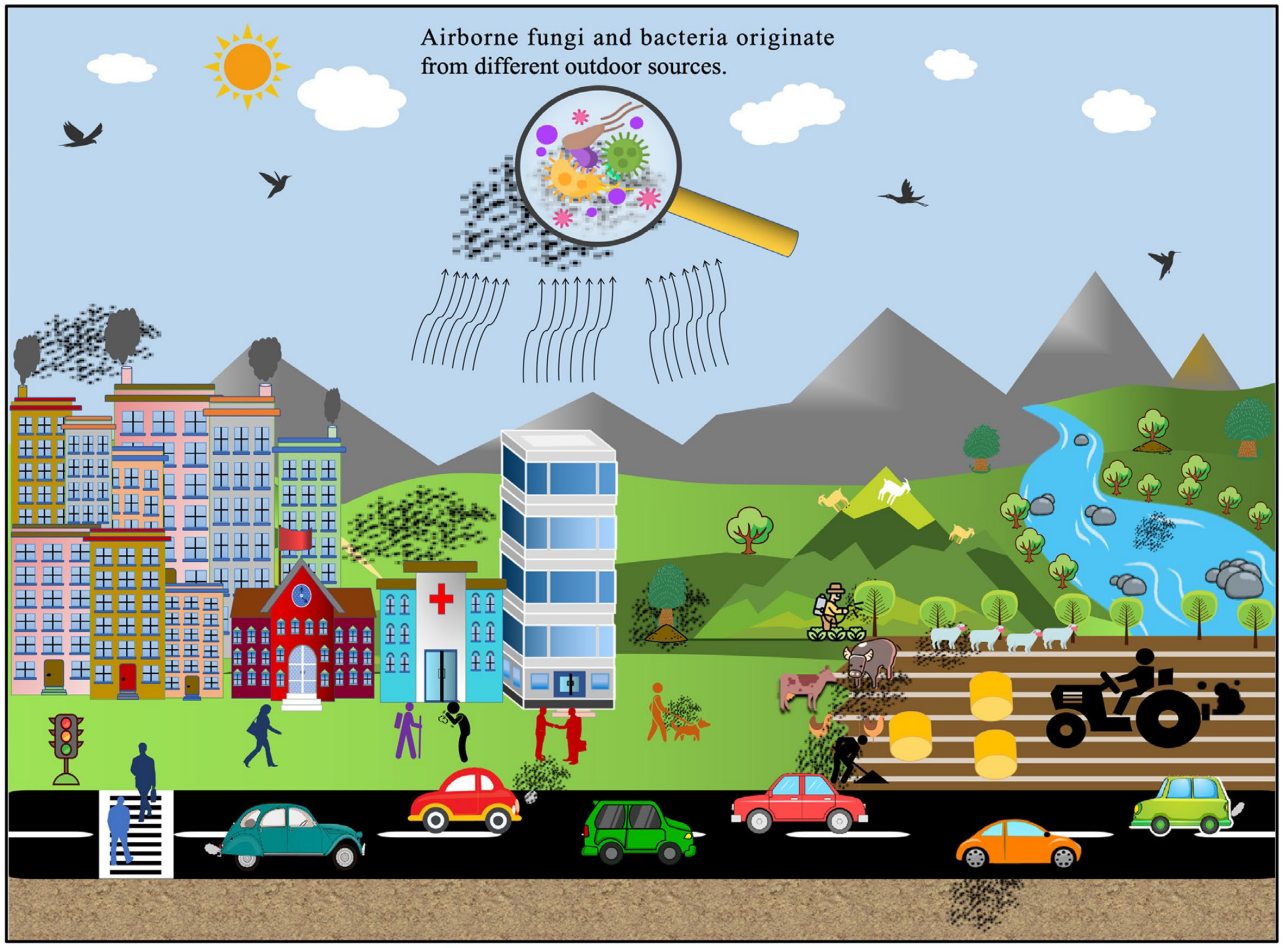
Diverse outdoor sources contributing to the presence of airborne fungi and bacteria.

Both outdoor urban and rural areas and indoor air environments, such as homes, offices, hospitals, schools, subway stations, etc., may represent reservoirs for a wide range of microorganisms, particularly fungi and bacteria (Figure 1; Tables 2, 3). These microbes have the potential to impact the overall ambient air quality and pose significant health hazards to human individuals within the above-mentioned places (Simon-Nobbe et al., 2008; Fernstrom and Goldblatt, 2013; Yuan et al., 2022). Numerous global research efforts have been directed toward investigating airborne microbes in various indoor and outdoor settings. These studies have revealed that the diversity of microbes detected may vary depending on various influencing factors, such as the investigated places or regions, sampling methods, seasonal changes, and specific environmental conditions (Adhikari et al., 2004; Fleischer et al., 2006; Wei et al., 2019; Chegini et al., 2020; Núñez and García, 2023). For instance, in a study conducted at a hospital in Madrid, Spain, it was reported that *Sphingomonas*, *Massilia*, *Hymenobacter*, *Streptomyces*, and *Methylobacterium*-*Methylorubrum* were the prevalent isolated bacterial genera, while in terms of fungi, the dominant genera were *Alternaria*, *Cladosporium*, *Penicillium*, and *Filobasidium* (Núñez and García, 2023). In contrast, at a university hospital in Turkey, the prevalent airborne bacterial species isolated were *Staphylococcus* spp., while the frequently isolated fungal genera were *Cladosporium* and *Penicillium*. However, in general, the literature consistently reports *Alternaria*, *Cladosporium, Penicillium*, *Aspergillus*, and *Fusarium* as the most common identified fungal genera in the indoor and outdoor air environments (Tables 2, 3). Similarly, *Bacillus*, *Streptococcus*, *Micrococcus*, *Enterococcus*, and *Pseudomonas* have been consistently documented as the dominant bacterial genera (Tables 2, 3). For example, studies conducted in various regions, such as Ohio, United States (Adhikari et al., 2004), Hangzhou, China (Fang et al., 2019; Lou et al., 2012), Qingdao, China (Wang et al., 2020), Basrah, Iraq (Muhsin and Adlan, 2012), Helwan, Egypt (Abdel Hameed et al., 2009), Santiago de Compostela, Spain (Aira et al., 2007), and Nanjing, China (Wu et al., 2019), consistently reported *Alternaria*, *Cladosporium*, *Penicillium*, and *Aspergillus* as the predominant fungal genera in different outdoor and indoor air environments.

**Table 2:** Dominant airborne fungi and bacteria in various indoor environments.

Study’s region	Study’s year	Sampling site	Investigated microorganism	Dominant identified airborne microorganism	Reference
Beijing, China	2017	Hospitals	Fungi	*Aspergillus*	Tong et al. (2017)
Wroclaw, Poland	2006		Bacteria and Fungi	Bacteria: *Staphylococcus aureus*, *Enterococcus* spp., *Pseudomonas aeruginosa*, *Acinetobacter lwoffii* and *Alcaligenes faecalis*. Fungi: *Penicillium* spp. and *Cladosporium* spp.	Fleischer et al. (2006)
West-Chennai, India	2012			Bacteria: *Staphylococcus* spp., *Micrococcus* spp., *Enterobacter* and *Pseudomonas*. Fungi: *Aspergillus niger*	Sudharsanam et al. (2012)
Madrid, Spain	2023			Bacteria: *Sphingomonas*, *Streptomyces*, *Massilia*, *Hymenobacter*, *Streptomyces*, and *Methylobacterium-Methylorubrum*. Fungi: *Cladosporium*, *Alternaria*, *Filobasidium* or *Penicillium*.	Núñez and García (2023)
Edirne, Turkey	2002			Bacteria: *Staphylococcus* spp. Fungi: *Cladosporium* and *Penicillium*.	Sarıca et al. (2002)
Tehran, Iran	2020	kindergarten		Bacteria: *Bacillus* spp., *Staphylococcus aureus*, *Micrococcus* spp., *Staphylococcus epidermidis*, *Staphylococcus saprophyticus*, *Enterococcus* spp., and *Streptococcus* spp. Fungi: *Aspergillus terreus*, *Aspergillus flavus*, *Cladosporium* spp., *Penicillium* spp., *Rhodotorula* spp., *Ulocladium* spp., and *Alternaria* spp.	Chegini et al. (2020)
Singapore	2011	Food courts		Bacteria: *Staphylococcus*, *Pseudomonas*, *Alcaligenes*, and *Corynebacterium*. Fungi: *Penicillium*, *Aspergillus*, and *Cladosporium*.	Rajasekar and Balasubramanian (2011)
Singapore	2012	Residential apartment		Bacteria: *Staphylococcus* and *Micrococcus* Fungi: *Aspergillus* and *Penicillium*	Balasubramanian et al. (2012)
Xi’an, China	2010	Museum		Bacteria: *Staphylococcus*, *Arthrobacter*, *Bacillus*, *Pseudomonas*, and *Micrococcus*. Fungi: *Penicillium*, *Alternaria*, and *Aspergillus*	Chen et al. (2010)
Seoul, South Korea	2009	Feedstuff-manufacturing factories		Bacteria: *Staphylococcus* spp., *Micrococcus* spp., *Corynebacterium* spp., and *Bacillus* spp. Fungi: *Cladosporium* spp., *Penicillium* spp., and *Aspergillus* spp.	Kim et al. (2009)
Nanjing, China	2021	Buildings		Bacteria: *Acinetobacter lwoffii*, *Bacteroides fragilis*, and *Acinetobacter baumannii*. Fungi: *Candida albicans*, *Malassezia restricta*, and *Aspergillus flavus*.	Guo et al. (2021)
Tianjin, China	2022		Fungi	*Alternaria*, *Cladosporium*, and *Aspergillus*.	Lu et al. (2022)
Shanghai, Beijing, Changsha, Wuhan, Dalian and Harbin, China	2021			*Aspergillus* spp., *Cladosporium* spp., and *Penicillium* spp.	Fan et al. (2021)
Hangzhou, China	2012	University campus, including living area, dining area, teaching area, and office area		*Penicillium*, *Cladosporium*, *Alternaria*, and *Aspergillus*.	Lou et al. (2012)
Nanjing, China	2019	Utility tunnel		*Aspergillus*, *Cladosporium*, *Alternaria*, and *Penicillium*.	Wu et al. (2019)
Bydgoszcz, Poland	2019	Sports facilities		*Cladosporium*, *Penicillium*, *Fusarium* and *Acremonium*.	Małecka-Adamowicz et al. (2019)
Santiago de Compostela, Spain	2007	Cathedral of Santiago de Compostela		*Alternaria*, *Aspergillus*, *Cladosporium*, and *Penicillium*.	Aira et al. (2007)
Seoul, South Korea	2016	Subway stations		*Penicillium*, *Aspergillus*, *Cladosporium*, and *Mucor*.	Hwang et al. (2016)
Milan, Italy	2000			*Cladosporium*, *Penicillium*, *Epicoccum*, and *Alternaria*.	Picco and Rodolfi (2000)
Dalian and Beijing, China	2019	Children’s dwellings		*Penicillium*, *Cladosporium*, and *Aspergillus*.	Lv et al. (2019)
Beijing, China	2022	Packaging, office, composting, and downwind areas		*Cladosporium*, *Alternaria*, and *Aspergillus*.	Gao et al. (2022)

**Table 3:** Dominant airborne fungi and bacteria in various outdoor environments.

Study’s region	Study’s year	Sampling site	Investigated microorganism	dominant identifies microorganism	Reference
Ohio, USA	2004	Agricultural environments	Fungi	*Cladosporium*, *Aspergillus*/*Penicillium*, *Epicoccum*, *Alternaria*	Adhikari et al. (2004)
Hangzhou, China	2019	Outdoor air environments		*Penicillium*, *Cladosporium*, *Alternaria*, *Aspergillus*, and *Trichoderma*.	Fang et al. (2019)
Lagos, Nigeria	2020			*Aspergillus* and *Penicillium*.	Odebode et al. (2020)
Basrah, Iraq	2012			*Cladosporium*, *Penicillium*, *Alternaria* and *Aspergillus*.	Muhsin and Adlan (2012)
Tehran, Iran	2014			*Aspergillus*, *Cladosporium*, *Penicillium*, and *Alternaria*.	Shams-Ghahfarokhi et al. (2014)
Rasht, Iran	2020		Bacteria and Fungi	Bacteria*: Bacillus* spp., *Staphylococcus aureus*, *Micrococcus* spp., *Staphylococcus epidermidis*, *Staphylococcus saprophyticus*, *Enterococcus* spp., and *Streptococcus* spp. Fungi*: Aspergillus terreus*, *Aspergillus flavus*, *Cladosporium* spp., *Penicillium* spp., *Rhodotorula* spp., *Ulocladium* spp., and *Alternaria* spp.	Chegini et al. (2020)
Kuopio, Finland	2008	Outdoor air of Urban and Rural Sites	Fungi	*Penicillium* and *Aspergillus*, *Cladosporium* spp.	Kaarakainen et al. (2008)
Colorado, USA	2013		Bacteria	Actinobacteria, Bacteroidetes, Firmicutes, and Proteobacteria	Bowers et al. (2013)
Qingdao, China	2020	Building rooftop	Fungi	*Cladosporium*, *Alternaria*, *Penicillium* and *Aspergillus*.	Wang et al. (2020)
Jinan, China	2017		Bacteria	*Lactococcus*, *Bacillus*, *Pseudomonas*, and *Psychrobacter*	Xu et al. (2017)
Hangzhou, China	2018			*Thiobacillus*, *Methylobacterium*, *Rubellimicrobium*, and *Paracoccus*	Liu et al. (2018)
Tianjin, China	2021	Urban outdoor areas	Fungi	*Alternaria*, *Cladosporium*, *Naganishia*, *Fusarium*, *Phoma*, and *Didymella*.	Nageen et al. (2021)
Beijing, China	2005			*Cladosporium*, non-sporing isolates, *Alternaria*, *Penicillium* and *Aspergillus*.	Fang et al. (2005)
Jinan, China	2019	Rural outdoor area	Bacteria and Fungi	Bacteria*: Acinetobacter*, *Cyanobacterium*, *Janthinobacterium*, and *Massilia*. Fungi*: Alternaria*, *Aspergillus*, *Cladosporium* and *Penicillium*	Wei et al. (2019)
Helwan, Egypt	2009	Industrial town of Helwan	Fungi	*Aspergillus*, *Penicillium*, *Alternaria*, and *Cladosporium*.	Abdel Hameed et al. (2009)
Tianjin, China	2023	Pedestrian bridges		*Alternaria*, *Cladosporium*, *Schizophyllum*, *Sporobolomyces*, and *Sporidiobolus*.	Al-Shaarani et al. (2023)

## 10 Conclusion and future directions

This review sheds light on the strong association between airborne fungi and bacteria and their impact on human health. Findings from a large number of studies clearly suggest that exposure to airborne microbes in various outdoor and indoor environments can have harmful effects on the human body, including infections, allergies, and toxic reactions, as well as many respiratory ailments. Therefore, it is important to prioritize heightened attention toward individuals who are exposed to airborne microbes, particularly toward vulnerable members of human populations such as children, elderly, patients with weakened immune systems, and various industry workers, such as those in the healthcare, construction, and agriculture sectors, who have consistently been reported as the most susceptible groups in different studies worldwide. It is also important to address the effect of different factors, such as regional differences, climate change, and other environmental parameters, on airborne microbial spread and growth. These factors could influence the risks associated with microbial presence in the air, emphasizing the need for future directions on air pollution monitoring and prevention. It would be necessary to develop new methods that allow real-time monitoring and easy detection of airborne microorganism outbreaks to facilitate the implementation of effective control measures. Additionally, it is crucial to highlight the need for future research focusing on the development of new technologies and strategies that could help in mitigating microbial hazards. This includes improving ventilation systems, exploring the use of antimicrobial coatings, especially in the healthcare sector, and promoting proper hygiene practices.
